# A Review of the Currently Available Antibody Therapy for the Treatment of Coronavirus Disease 2019 (COVID-19)

**DOI:** 10.3390/antib12010005

**Published:** 2023-01-11

**Authors:** Kristin Widyasari, Jinnam Kim

**Affiliations:** 1 Gyeongsang Institute of Health Sciences, Gyeongsang National University, Jinju 52727, Republic of Korea; 2 Major of Food Science & Nutrition, Pukyong National University, Busan 48513, Republic of Korea

**Keywords:** human coronavirus, SARS-CoV-2, COVID-19, monoclonal antibody, therapy

## Abstract

Monoclonal antibodies are a promising treatment for COVID-19. However, the emergence of SARS-CoV-2 variants raised concerns about these therapies’ efficacy and long-term viability. Studies reported several antibodies, that received authorization for COVID-19 treatment, are not effective against new variants or subvariants of SARS-CoV-2, hence their distribution has to be paused. Here, the authors reviewed the status of the currently available monoclonal antibodies for COVID-19 treatment, their potential as a therapeutic agent, and the challenges ahead. To address these issues, the authors presented general information on SARS-CoV-2 and how monoclonal antibodies work against SARS-CoV-2. The authors then focus on the antibodies that have been deployed for COVID-19 treatment and their current status, as well as the evidence supporting their potential as an early intervention against COVID-19. Lastly, the authors discussed some leading obstacles that hinder the development and administration of monoclonal antibodies for the treatment of COVID-19.

## 1. Introduction

Human coronaviruses (hCoVs) were first characterized in the 1960s when an infectious agent was found in organ culture from the respiratory tract of an adult with the common cold. The term coronavirus was given due to the crown-like appearance on its surface [1]. The coronaviruses comprise multiple strains of human and animal viruses that cause respiratory tract infections, which range from mild to lethal [2,3]. Members of coronavirus share similarities in their structure. They are enclosed in a lipid bilayer envelope protein that contains two or three glycoproteins, i.e., a matrix protein, a surface component, and a haemagglutinin esterase, which is found in several betacoronaviruses. The RNA genome is surrounded by a nucleoprotein and, together, they appear as a coiled tubular helix within the lipid bilayer envelope [4]. Genes of the major structural proteins in all coronaviruses occur in the 5′ to 3′ order as spike protein (S), an envelope protein (E), matrix protein (M), and nucleoprotein (N) [5].

Infection with hCoV occurs more often during winter and spring in temperate climates [6,7,8]. During these past 20 years, numerous hCoVs have been identified; three among them, named severe acute respiratory syndrome coronavirus (SARS-CoV), the Middle East respiratory syndrome coronavirus (MERS-CoV), and severe acute respiratory syndrome coronavirus-2 (SARS-CoV-2), are noticeably more contagious than other hCoVs and have caused significant mortality worldwide [9,10,11]. Infection by SARS-CoV, MERS-CoV, and SARS-CoV-2 results in acute lung injury, acute respiratory distress syndrome, septic shock, and multiple organ failure [12,13]. However, the transmission rate of SARS-CoV and MERS-CoV is lower and they were easily contained compared to SARS-CoV-2 [14].

Vaccination remains the primary option for the prevention of coronavirus diseases, while antibody therapies may be still viewed as an “add-on” treatment. Despite this, the rapid advancement of antibody research and development offers different insights and renewed optimism for the use of antibody therapy for the treatment of coronavirus disease. This review presents an overview of the currently available antibody therapies, as well as the challenges in addressing the coronavirus disease, particularly COVID-19, based on published findings.

## 2. SARS-CoV-2

**Figure 1:**
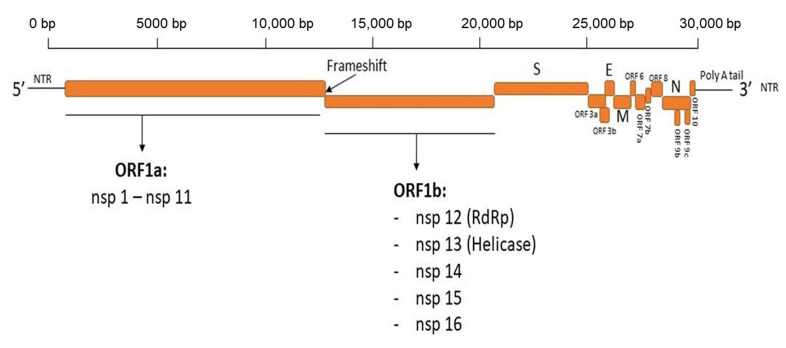
Diagram depicting the genome organization of SARS-CoV-2. The genome of SARS-CoV-2, with a size of ~30 Kb, encodes 4 structural proteins, 16 non-structural proteins (nsps), and 6 accessory proteins. The structural proteins, including spike glycoprotein (S), nucleocapsid (N), membrane (M), and envelope (E) proteins, are important for virus assembly and infection and are the target for the development of vaccines and therapeutics for COVID-19.

The novel severe acute respiratory syndrome coronavirus (SARS-CoV-2), the causative agent of COVID-19, was first identified in Wuhan, Hubei Province, China at the end of 2019. SARS-CoV-2 is a member of the family *Coronaviridae*, the subfamily *Orthocoronaviridae*, and the genus *betacoronavirus* [15,16]. SARS-CoV-2 is a positive strand ssRNA animal virus; the genome size of SARS-CoV-2 varies from 29.8 kb to 29.9 kb [17]. The SARS-CoV-2 genome is non-segmented and has been reported to share a high similarity in the sequence identity for essential enzymes and structural proteins; up to 82% with SARS-CoV and about >90% with MERS [18]. The genome of SARS-CoV-2 encodes both structural proteins, which are responsible for viral assembly and the maturation of viral particles, and non-structural proteins that play crucial roles in viral RNA replication and immune evasion, including aiding viral infection and transmission in host cells [19,20,21] (Figure 1).

Among the SARS-CoV-2 viral proteins, the spike protein (S), envelope (E), membrane (M), and nucleocapsid (N) proteins are the main target of the structural proteins-based therapeutics for SARS-CoV-2 [22,23,24] (Table 1).

SARS-CoV-2 continuously evolves as mutations occur during its genome replication [39]. To date, multiple variants and subvariants of SARS-CoV-2 with more than a million sequences have been made public and are being updated continuously, on a real-time basis, through the Global Initiative on Sharing All Influenza Data (GISAID) [40,41]. The changes in the genetic codes of SARS-CoV-2 may affect the virus’ characteristics, including transmissibility, antigenicity, infectivity, and severity [42].

The attachment of the SARS-CoV-2 to the host cell surface is the “key” for the virus to gain entry to the host cells. The spike protein (S) mediates the attachment of the SARS-CoV-2 to the host cell by binding into the human angiotensin-converting enzyme 2 (hACE2) receptor through its receptor-binding domain (RBD) [43]. The S is also the practical target for neutralizing antibodies [44]. Thus, for this reason, S is the major target for the development of therapeutic agents or vaccines, which are the primary option for the prevention of COVID-19. Among SARS-CoV-2 proteins, the gene that encodes S is the most notable region where the mutations occurred. To date, more than 4000 mutations in the gene encoding the S gene have been identified [45]. Among these mutations, some may not give any phenotype effects [41], but some may change the virus’ characteristics, including the antigenicity of the S, hence, resulting in viral adaptability and the emergence of variants that can evade neutralization by vaccine-induce immunity, natural immunity or monoclonal antibodies [46,47,48] (Table 2). The variations that occur in the RBD area of S enhance the binding affinity of RBD with hACE2, thus reducing the neutralization activity by the neutralizing antibodies or nanobodies [49].

The continuous changes in the predominant variants of SARS-CoV-2 have become a concern worldwide. By October 2021, the delta was the dominating variant, which reached almost 90% of all viral sequences submitted to GISAID [69]. However, currently, the predominant variant circulating globally is omicron, comprising >98% of the viral sequences shared on GISAID after February 2022 [69]. Since omicron was designated as the variant of concern (VOC) on 26 November 2021 [69], multiple subvariants of omicron have been reported. Those subvariants include B.1.1.529, BA.1, BA.1.1, BA.2, BA.3, BA.4, and BA.5 [70]. Recently, the new subvariants of omicron, named BQ.1 and BQ.1.1, were reported to have become the dominant subvariants in the U.S. [71], and the XBB subvariant has become particularly prevalent in the countries of South East Asia [72]. Thus, with the ongoing emergence of VOCs with higher transmissibility and pathogenicity, researchers around the world are working around the clock to address the urgent demand for effective therapeutic and preventive measures with a broad-spectrum against SARS-CoV-2, particularly the omicron variant and its subvariants.

## 3. Neutralizing Monoclonal Antibodies against SARS-CoV-2

The COVID-19 pandemic caused a devastating impact on many sectors of human life, caused millions of people to lose their lives and became an exceptional public health crisis that urgently demands the development of timely and accurate therapeutics. Over the past years, during the COVID-19 pandemic, tremendous research efforts and financial resources have been dedicated to the development of diagnostic, prophylactic, and therapeutic measures for COVID-19 [29,73,74].

Unlike the vaccine-derived immunity that develops over time before effectively giving protection against SARS-CoV-2, the administration of monoclonal antibodies as a therapeutic agent may give immediate and passive immunotherapy, with the potential to reduce disease progression immediately after the administration, and also the potential to reduce the severity of the disease [75]. These monoclonal antibodies are a novel class of antiviral intervention that can ‘neutralize’ SARS-CoV-2 in infected patients. Thus, antibody therapy has been suggested as a promising option to prevent the development of severe infection of COVID-19 in high-risk individuals.

Neutralizing monoclonal antibodies are recombinant proteins that are derived from B cells. The molecule of a monoclonal antibody is comprised of four polypeptide chains, with two identical heavy and light chains. Disulfide linkages connect all the chains to form a “Y” shaped tetramer. High-throughput screening of B cells from convalescent patients, vaccinated individuals or humanized mice permits the identification of IgG class antibodies with specificity and affinity to bind into virus surface protein and block entry of the virus into the healthy cells [76,77]. The affinity of the Fab region in the antibody is critical for binding to the target antigen, thus determining the specificity of the antibody. The high specificity and affinity of antibodies ensure a more precise action on the target (antigen), so thus the virus can be directly neutralized or attacked by the component of the immune system. According to Taylor et.al, during the viral infection, antibodies can work either by preventing the binding/fusion of virion with the target cells (neutralization), or by opsonizing the virion, or infected cells for phagocytic uptake [78]. The neutralization mechanism of the antibody can vary, including direct blocking of viral entry, antibodies-mediated effector functions, or inactivating the viral entry glycoprotein [79].

**Figure 2:**
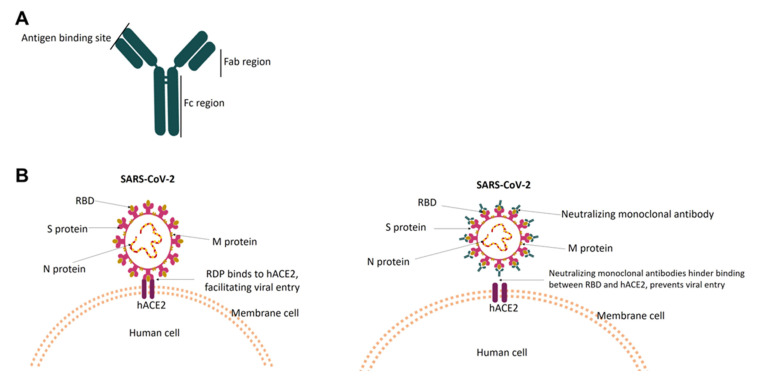
Diagram representing the structure of the monoclonal antibody, and the neutralizing mechanism. In (**A**), the monoclonal antibody is comprised of chains that are connected by disulfide linkage to form a “Y” shape. The region that binds to the target antigen is indicated. In (**B**), the RBD binds to hACE, which facilitates viral entry into the cells (**left**). Monoclonal antibodies disrupt the RBD–hACE interaction by targeting the RBD (antigen), thus hindering viral entry into the cells (**right**).

As a COVID-19 therapeutic agent, the neutralizing monoclonal antibody works by specifically targeting the RBD in the S of SARS-CoV-2, thus inhibiting the RBD–hACE2 interaction. Failure of RBD to bind with hACE2 results in the inability of SARS-CoV2 to enter neighboring cells [73,80], hence, giving protection against reinfection with SARS-CoV-2 or preventing the disease’s progression (Figure 2).

## 4. Currently Available Monoclonal Antibodies for COVID-19 Treatment

As part of COVID-19 control and prevention, the U.S. Food and Drug Administration issued an emergency use authorization (EUA) for the neutralizing monoclonal antibodies: bamlanivimab and etesevimab to be administered together on 9 February 2021 [81]. On November 9, the investigational monoclonal antibody bamlanivimab received EUA to be administered alone [82], and after ten days, on 21 November 2021, casirivimab and imdevimad also received a EUA to be administered together for the treatment of mild to moderate COVID-19 in adults and pediatric patients with positive infection of SARS-CoV-2 [33]. Following approval of these monoclonal antibodies as a COVID-19 treatment, multiple other monoclonal antibodies received a EUA from the FDA, or authorization in certain countries, to be administered for the treatment of COVID-19 in vulnerable populations (Table 3).

The administration of some neutralizing monoclonal antibodies, either as a combination of two antibodies or as a single treatment in individuals with mild to moderate COVID-19 infection, worked well in the early pandemic [32,100,101,102]. However, with the emergence of new variants of SARS-CoV-2, the treatment using some antibodies, such as bamlanivimab is no longer effective against SARS-CoV-2′s variants. Bamlanivimab has a limited effect against the beta and gamma variants and is not effective against the delta and omicron variants [103,104,105]. Similarly, etesevimab was also reported to not neutralize the omicron, beta, or gamma variants, even at the FRNT_50_ > 50,000 ng per milliliter. Meanwhile, imdevimab showed high neutralizing activity against the beta and gamma variants but is not effective against the omicron variant. The administration of casirivimab demonstrated its ability to neutralize beta, gamma, and omicron with an FRNT_50_ value of 187.69 to 14,110.70 ng per milliliter [105]. Correspondingly, tixagevimab, cilgavimab, and sotrovimab were reported to retain neutralization activity against beta, gamma, and omicron [105,106,107], with an FRNT_50_ value for omicron being higher by multiple folds compared to the other variants.

Given that omicron is currently the predominant VOC worldwide, it is essential to safeguard vulnerable populations by administering neutralizing monoclonal antibodies that can neutralize the omicron variant and its subvariants. Among the available neutralizing monoclonal antibodies for COVID-19 treatment, evusheld and bebtelovimab demonstrated effectiveness against the omicron variants, thus remain authorized in the U.S. Additionally, some studies reported candidates of neutralizing monoclonal antibodies that potentially neutralize a broad-spectrum of SARS-CoV-2, including omicron and its subvariants. Hence, these monoclonal antibodies may become alternative therapeutics to answer the current challenges of SARS-CoV-2.

### 4.1. Evusheld (Combination of Tixagevimab and Cilgavimab)

Evusheld, developed by AstraZeneca (Cambridge, UK), is a combination of two long-acting antibodies: tixagevimab and cilgavimab, derived from B-cells donated by convalescent patients after SARS-CoV-2 infection, which have been optimized with a half-life extension, and a reduction in Fc effector function and complement C1q binding [108,109]. Results from the PROVENT phase III trial revealed that evusheld was able to give protection up to months after the administration of a single dose [110]. The FDA issued a EUA for evusheld for pre-exposure prophylaxis and the treatment of symptomatic disease caused by SARS-CoV-2 in persons 12 years and older, who have either a history of severe or moderate allergy to COVID-19 vaccines [84]. The recommended single dose of evusheld for prevention against COVID-19 is 600 mg; comprising 300 mg of tixagevimab and 300 mg of cilgavimab, administered as separate sequential intramuscular injections every six months [111]. This recommended dosage is double the initial recommendation (150 mg each of tixagevimab and cilgavimab) [108,112]. The dosing regimen was revised due to updated data that indicated that the originally recommended dosage was less effective against the omicron variant and its subvariants. The latest data indicate that a higher dose of evusheld is likely to prevent infection by the omicron subvariants BA.1 and BA.1.1 [111].

### 4.2. Bebtelovimab

Bebtelovimab is a recombinant neutralizing human monoclonal antibody developed by AbCellera (Vancouver, Canada) and Eli Lilly (Indianapolis, USA) as a treatment for mild-to-moderate COVID-19 in high-risk adults and children (12 years and older). The FDA issued a EUA for the emergency use of bebtelovimab on 11 February 2022 [30]. Unlike other preexisting human monoclonal antibodies, which are mostly less effective against the omicron variant, bebtelovimab showed a remarkably preserved activity against all SARS-CoV-2 variants, including two subvariants of omicron: BA.4 and BA.5 [29,113,114]. SARS-CoV-2 variants with mutations at the amino acid positions 417, 439, 452, 484, and 501, greatly affect the in vitro binding of antibodies, thus reducing the effectivity of preexisting anti-SARS-CoV-2 monoclonal antibodies and vaccines [52]. Nevertheless, bebtelovimab binds to an epitope that is largely distinct from the mutations identified from the emerged variants, including mutations that greatly reduce the effectiveness of preexisting anti-SARS-CoV-2 monoclonal antibodies and vaccines, and retain binding and neutralization activity against variants of SARS-CoV-2 [29]. Currently, the clinical data on bebtelovimab’s efficacy are limited. However, the results from trials showed that bebtelovimab appears safe and able to decrease the risk of hospitalization and death. Bebtelovimab may be given when other treatment options for COVID-19 are unavailable or inappropriate. According to the FDA, bebtelovimab should be given as soon as possible after confirmation of SARS-CoV-2 infection and within 7 days of symptom onset with the recommended dosage for administration being 175 mg [115,116].

### 4.3. Bispecific Antibodies

Apart from the recombinant monoclonal antibodies that received a EUA for emergency use by the FDA, multiple antibodies are currently under investigation and have been reported to have broad neutralizing activity against SARS-CoV-2 variants, including the currently predominant variant. One among those antibodies is the CoV-X2, which is a bispecific IgG1-like molecule that was developed based on antibodies (C121 and C135) derived from donors who had recovered from COVID-19. The CoV-X2 showed simultaneous binds to two independent sites on the RBD and prevented spike binding to the ACE2. This antibody was also reported to be able to neutralize not only the SARS-CoV-2 wild type but also another variant of concern, as well as the parental monoclonal antibodies escape mutants [117]. Another IgG-like bispecific antibody (BsAb), developed by Chang et.al [118], also demonstrated potent and synergistic neutralization against circulating SARS-CoV-2 variants of concern. Administration of BsAb as a post-infection treatment in golden hamsters and as a prophylaxis in mice demonstrated enhanced binding and distinct synergistic effects on the neutralizing activity against variants of concerns [105]. Thus, the bispecific antibodies may be our new hope for an antibody therapy that can maintain its effectiveness against new variants of SARS-CoV-2.

### 4.4. Antibodies That Alleviate the Harmful Effect of an Over-Stimulated Host Immune Response

The therapeutic use of antibodies is relying on the fact that antibodies prevent the interaction of the S protein and hACE2, thus preventing the entry of SARS-CoV-2 into neighboring cells. However, some monoclonal antibodies were reported to be able to alleviate an over-stimulated host immune response (cytokine storm) due to COVID-19. Cytokine storms are defined as an acute overproduction and uncontrolled release of pro-inflammatory markers [119]. Three important cytokines in the interleukin 1 (IL-1) family are especially relevant to cytokine storms, i.e., IL-1β, IL-18, and IL-33. A study reported that blocking IL-1β can potentially prevent a cytokine storm [120]. Given that COVID-19 can trigger cytokine storms in pulmonary tissues through hyperactivation of the immune system [121], the appropriate therapy is required to alleviate the damage caused by this over-stimulated host immune response. Canakinumab is a human anti-IL-1β monoclonal antibody that directly neutralizes IL-1β [122]. Studies have reported that the administration of canakinumab leads to a reduction in inflammation and a long-lasting improvement in oxygenation levels in the absence of any severe adverse events of COVID-19 [123]. A similar monoclonal antibody that showed its potential in managing a cytokine storm due to COVID-19 is adalimumab [124]. Adalimumab is a human monoclonal antibody that targets tumor necrosis factor alpha (TNR-α), a cytokine that has a pleiotropic effect on various cell types and plays an important role in cytokine storms [125]. The potential role of anti-TNFα or anti-inflammation antibodies in treating COVID-19 is strictly linked to the control of the pathogenetic mechanisms during viral infection [126]; hence, the therapeutic use of monoclonal antibodies that target the cytokines that are relevant to a cytokine storm may become a promising approach for the management of acute respiratory distress syndrome in patients with COVID-19.

## 5. Challenges to the Use of Antibody Therapies for COVID-19

Throughout the history of pandemics, apart from vaccines or convalescent plasma therapy, the use of therapeutic monoclonal antibodies is viewed as an alternative treatment to reduce mortality. The uses of monoclonal antibody therapies are mostly designated for populations that develop allergies or respond weakly to vaccination, i.e., immunocompromised patients [127], or other high-risk populations.

A great drawback of these therapies is that monoclonal antibodies have high specificity and affinity, thus a small change (mutation) in epitope frequently renders a failure of the antibody in neutralizing the target. Currently, available monoclonal antibodies are most likely targeting RBD and NTD, which are prone to mutate. Hence, these antibodies are more likely to lose their neutralizing activities against a newly emerging variant of SARS-CoV-2. A monoclonal antibody that targets the conserved viral epitopes is important for the development of broad-spectrum antibody therapies. The epitopes in the S2 subunit are reported to be more conserved than those in the S1 subunit [128]. Hence, the epitopes in the S2 subunit may become a potential target for the development of broad-spectrum antibodies. However, despite having a broader neutralizing spectrum, the S2 antibodies are much less potent than the one that targets RBD [129]. Therefore, balancing the breadth and efficacy of neutralizing antibodies is crucial during the selection of candidate antibodies for COVID-19 treatment.

The identification of conserved epitopes, as well as the development of neutralizing monoclonal antibodies that have a broad-neutralizing activity, is not only conducted by a major stakeholder, but also by many researchers all around the world. However, many laboratories are not certified to work with human pathogens, making the efficacy assessment of developed antibodies meet a bottleneck [127]. Using a pseudotyped virus-based assay for assessment may be an alternative, to reduce the risk of accidents while using a living virus. However, the development, distribution, and implementation of a pseudotyped virus-based assay may take some time before being readily available for all variants and subvariants of SARS-CoV-2; hence, becoming another obstacle to the rapid development of neutralizing monoclonal antibodies.

In addition, regulatory approval may become another limitation. The rapid development and distribution of monoclonal antibodies are critical during a public health crisis. However, in some cases, although some monoclonal antibodies for COVID-19 have already received authorization for emergency use, there are still delays or lags in distribution and administration in some regions due to certain policies [130]. This situation may undoubtedly increase the risk of infection, or worse, mortality in vulnerable and high-risk populations.

Another concern regarding the uses of neutralizing monoclonal antibodies for COVID-19 treatment is their shelf-life. The shelf-life of monoclonal antibodies is relatively short, thus, antibodies for COVID-19 treatment that are no longer authorized may have to be discarded, and this equals a big financial loss. To address this issue, the FDA announced the extension of some COVID-19 monoclonal antibodies: REGEN-COV from 24 to 30 months [90], sotrovimab, and bamlanivimab from 12 to 24 months [131,132], with the expectation that these antibodies will be effective against future SARS-CoV-2 variants that may be susceptible to these antibodies. However, because future events are unpredictable, there is still a possibility that these batches of antibodies will also end up being discarded. Nevertheless, despite all these concerns and limitations, the development of monoclonal antibodies as an alternative COVID-19 treatment is necessary to provide access to lifesaving therapies.

## 6. Conclusions

Neutralizing monoclonal antibodies are a promising prophylactic and therapeutic treatment for COVID-19. However, the effectiveness and future of currently available neutralizing monoclonal antibodies have been questioned by the emergence of SARS-CoV-2 variants and their subvariants. Characterization of the conserved epitopes in SARS-CoV-2 and the development of monoclonal antibodies that directly target these epitopes, as well as the crucial cytokines involved in SARS-CoV-2 pathogenicity, may be an alternative answer for the development of future monoclonal antibody therapies with more breadth and high effectiveness against SARS-CoV-2 variants.

## Figures and Tables

**Table 1:** Available drugs and non-vaccine therapeutics, with their target proteins, up to November 2022.

Therapeutics	Type	Target Protein (SARS-CoV-2)	References
Nirmatrelvir with Ritonavir (Paxlovid)	Antiviral drug	Viral protease (M^pro^)	[25,26]
Remdesivir (Veklury)	Antiviral drug	RdRp	[27,28]
Bebtelovimab	Monoclonal antibody	Spike protein (RBD)	[29,30]
Molnupiravir (Lagevrio)	Antiviral drug	RdRp	[31]
Bamlavinivmab with etesevimab	Monoclonal antibody	Surface spike glycoprotein	[32]
Casirivimab with imdevimab	Monoclonal antibody	Spike protein	[33,34]
Sotrovimab	Monoclonal antibody	Spike protein	[35,36]
Tixagevimab with cilgavimab	Monoclonal antibody	Spike protein	[37,38]

Abbreviation: M^pro^, main protease; RdRp, RNA-dependent RNA polymerase; RBD, receptor binding domain.

**Table 2:** Major mutations in the SARS-CoV-2 protein and its impacts.

Mutations	Type of Mutation and Region	Impact	References
D614G	Amino acid substitution within receptor-binding motif (RBM)	- Increases infectivity. - Increases transmissibility.	[50,51]
N439K	Amino acid substitution within receptor-binding motif (RBM)	- Enhances the binding affinity for the ACE2 receptor. - Reduces the neutralizing activity of some monoclonal antibodies (mAbs) and polyclonal antibodies.	[52]
Y453F	Amino acid substitution within receptor-binding motif (RBM)	- Increases ACE2-binding affinity. - Enhances transmission capacity.	[53,54]
Δ69–70	Amino acid deletion in the N-terminal domain (NTD) of the spike protein	- Affects the network of NTD loops. - Increases transmission capacity.	[55,56]
N501Y	Amino acid substitution within the RBD	- Strengthens S protein binding to receptor ACE2. - Facilitates immune escape (antibody).	[42,57]
E484K	Amino acid substitution within the RBD	Reduces the neutralizing activity of antibodies.	[58,59]
K417N	Amino acid substitution in the spike protein	- Increases the interaction with hACE2. - May abolished the antibody effect.	[60]
K444 Q/R/N	Amino acid substitution within the RBD	Reduces the neutralizing activity of antibodies.	[61]
V445E	Amino acid substitution within the RBD	Reduces the neutralizing activity of antibodies.	[42,61]
K150 T/Q/R/E	Amino acid substitution in NTD of the spike protein	Reduces the neutralizing activity of antibodies.	[61,62]
N148S	Amino acid substitution in NTD of the spike protein	Reduces the neutralizing activity of antibodies.	[61]
L452R	Amino acid substitution within the RBD	- Increases infectivity. - Increases viral fusogenicity. - Facilitates escape antibodies.	[63]
P681R	Amino acid substitution in the spike protein	- Enhances viral fusogenicity. - Increases viral pathogenicity.	[64]
F486V	Amino acid substitution within the RBD	- Facilitates escape from certain class 1 and 2 antibodies.	[65]
N460K	Amino acid substitution in the spike protein	- Enhances S processing. - Enhances the resistance to neutralizing antibodies.	[66]
R346T	Amino acid substitution in the spike protein	- Increases viral prevalence. - Increases the ability to evade neutralizing antibodies.	[67,68]

**Table 3:** List of antibodies that received authorization for COVID-19 treatment up to November 2022.

No	Antibodies Name	Current Status	References
1	Bebtelovimab	Remain authorized in the U.S. until further notice by the FDA.	[30,83]
2	Tixagevimab with cilgavimab	Remain authorized with the recommendation of repeat dosing every six months with a dose of 300 mg of tixagevimab and 300 mg of Cilgavimab.	[84,85]
3	Sotrovimab	Since 5 April 2022, no longer authorized in any U.S. region; approved in Australia, the UK, and the EU.	[86,87,88]
4	Bamlanivimab with etesevimab	Pausing all distribution.	[81,89]
5	Casirivimab with imdevimab	Currently not authorized in any U.S. region; however, it is recommended to be retained for future SARS-CoV-2 variants that may be susceptible.	[33,88,90]
6	Amubarvimab/romlusevimab	Approved in China.	[88]
7	Regdanvimab (CT-P59)	Approved in the Republic of Korea and the EU.	[88]
8	Ronapreve	Approved in Japan, the UK, the EU, and Australia.	[88]
9	F61	Approved for clinical trials in China.	[91]
10	Tocilizumab	Authorized for emergency use in June 2021.	[92,93]
11	Sarilumab	Clinical trial phase 3.	[94]
12	Adalimumab	Clinical trial phase 3.	[95]
13	Canakinumab	Clinical trial phase 3.	[96]
14	Ravulizumab	Completed clinical phase 3; recruiting phase 4 trials.	[97,98]
15	Lenzilumab	Clinical trial phase 3.	[99]

Abbreviation: EUA, emergency use authorization; FDA, U.S. Food and Drug Administration; mg, milligram.

## Data Availability

Not applicable.
